# A Novel Compound Heterozygous Mutation of HSD17B3 Gene Identified in a Patient With 46,XY Difference of Sexual Development

**DOI:** 10.1016/j.esxm.2022.100522

**Published:** 2022-05-17

**Authors:** Carlotta Cocchetti, Fulvia Baldinotti, Alessia Romani, Jiska Ristori, Francesca Mazzoli, Linda Vignozzi, Mario Maggi, Alessandra Daphne Fisher

**Affiliations:** 1 Andrology, Women's Endocrinology and Gender Incongruence Unit, Florence University Hospital, Florence, Italy; 2 Laboratory of Molecular genetics, Azienda Ospedaliero-Universitaria Pisana, Pisa, Italy; 3 Department of Experimental and Clinical Biomedical Sciences “Mario Serio”, University of Florence, Florence, Italy

**Keywords:** 17β-Hydroxysteroid Dehydrogenase Type 3, Differences of Sexual Development, DSD, Gender Identity, GnRH Analogues

## Abstract

**Introduction:**

Deficiency of the 17β-hydroxysteroid dehydrogenase type 3 (17 β-HSD3) is a rare autosomal recessive 46,XY Difference of sex development (DSD), resulting from pathogenetic variants in the HSD17B3 gene, which lead to absent or reduced ability to convert Δ4-androstenedione to testosterone in the fetal testes.

**Aim:**

This study aimed to present the clinical and genetic characteristics of an Italian patient receiving a diagnosis of 17 β-HSD3 deficiency in adulthood. The patient was raised as female and underwent early surgical interventions to correct virilized genitalia, leading to a significant sexual distress.

**Methods:**

At the time of the referral, a 20-gene Next Generation Sequencing custom-panel for DSD was performed on patient's genomic DNA.

**Results:**

A novel compound heterozygous mutation in HSD17B3 gene was identified, detecting a new variant (c.257_265delAGGCCATTG, p.)

**Conclusion:**

Novel genotype causing 17 β-HSD3 deficiency is presented. Furthermore, the patient's clinical history stresses the importance to actively involve these individuals in the decision-making process avoiding surgical intervention when the patient is not able to give fully informed consent.

**Cocchetti C, Baldinotti F, Romani A, et al. A Novel Compound Heterozygous Mutation of HSD17B3 Gene Identified in a Patient With 46,XY Difference of Sexual Development. Sex Med 2022;10:100522.**

## INTRODUCTION

Steroid 17β-hydroxysteroid dehydrogenase type 3 (17 β-HSD3) is a crucial enzyme involved in testicular steroidogenesis, which catalyses the conversion of Δ4-androstenedione (Δ4-A) into testosterone (T).^1^ Pathogenetic variants of the *HSD17B3* gene lead to 17 β-HSD3 deficiency, a rare autosomal recessive cause of 46,XY Differences of Sexual Development (DSD).^2^ The HSD17B3 gene (OMIM 605573), mapped to chromosome 9q22, consists of 11 exons and encodes the aforementioned HSD17B3 enzyme of 310 amino acids.^2^ To date, only 68 mutations have been identified in this gene (http://www.hgmd.cf.ac.uk/ac/gene, HGMD Professional 2020.4). The defect of the last step of testicular steroidogenesis, leading to decreased T levels during foetal development, results in under virilized genitalia in 46,XY newborns.^3^, ^4^, ^5^ Clinical phenotype may range from normal female appearance of external genitalia to under virilized male genitalia, including micro-phallus with hypospadias.^3^^,^^4^ Due to normal anti-Müllerian hormone secretion, patients lack Müllerian structures, whereas Wolffian derivatives are often normally developed and testes located in the inguinal canal.^3^^,^^4^ Since in most cases affected new borns present female external genitalia, female gender is usually assigned at birth.^6^^,^^7^ However, if gonadectomy is not performed, virilization occurs at puberty with deepening in voice, development of male body habitus and body hair distribution and clitoris enlargement.^4^^,^^8^

Considering the rarity and clinical heterogeneity of this 46,XY DSD, diagnosis may be challenging and confirmed only in adulthood. The main biochemical characteristic of 17 β-HSD3 deficiency is represented by reduced T: Δ4-A ratio post-hCG stimulation (< 0.8).^9^ However, the endocrine evaluation may be difficult to interpret, thus direct genetic testing is commonly used to confirm the diagnosis.

We here describe the clinical and genetic findings in an Italian patient with 17 β-HSD3 deficiency diagnosed in adulthood. In particular, the mutation analysis identified a novel compound heterozygous mutation in HSD17B3 gene.

## MATERIALS AND METHODS

The patient was born at term in 1963 from non-consanguineous Italian parents without family history of ambiguous genitalia. Given the female appearance of external genitalia, she was assigned female at birth and raised as girl. At puberty, primary amenorrhea occurred as well as deepening of voice, clitoris enlargement and pubarche in the absence of breast development. For this reason, at the age of 15 she was first referred to clinical attention. Physical examination revealed severe clitoral hypertrophy (4.5 cm of length) with separate vaginal and urethral openings. Bilateral palpable testes were identified in the inguinal region. Gonadal biopsy was performed, revealing the presence of testicular tissue with interstitial cells hypertrophy and undifferentiated elements in the tubules. Chromosomal analysis on peripheral blood cells showed a 46, XY karyotype, but no further investigations were performed. The patient underwent surgery (gonadectomy, clitoridotomy and vaginoplasty) in 1979 when she was 16 years old. After that, estrogen replacement treatment was started.

The patient referred to *** gender clinic when she was 55 years old. When asked about her gender identity, she reported a life-long identification with the female gender. Besides, she reported a significant distress towards the aspect of her external genitalia – especially the complete absence of the clitoris – and this leads her to avoid intimacy with her partner. Therefore, she was referred to clitoral reconstruction surgery, which partially reduced the perceived body uneasiness.

At the same time, in order to make a diagnosis, genomic DNA was extracted from peripheral blood samples according to standard procedures, and a 20-gene Next Generation Sequencing (NGS) custom-panel for DSD was performed. Target enrichment was done by SureSelect XT (Agilent Technologies, Santa Clara, CA, USA) according to the manufacturer's instructions. A template library was prepared and sequenced using MiSeq Illumina platform (Illumina, San Diego, CA, United States). Annotation and filtering of variants were performed with Variant Interpreter platform; the NGS coverage was analyzed in detail using Integrative Genome Viewer version 2.3. Variants and regions with a depth coverage below 50 were confirmed by Sanger sequencing - using previously described procedures - and were checked for previously reported causative mutations in published works and variant databases: (i) Human Gene Mutation Database (HGMD), and (ii) Leiden Open Variation Database (LOVD). Variants were classified following the American College of Medical Genetics (ACMG) and genomics recommendations^10^ using population frequency databases (dbSNP, ExAC, and GnomAD) and in silico tools including PolyPhen, SIFT, Align GVGD, DANN, FATHMM, Mutation Assessor, and MutationTaster.

Written informed consent was obtained from the patient for publication of this case report.

## RESULTS

The DSD custom NGS panel detected the presence of two genetic variants in the HSD17B3 gene: c.239G>A, p.(Arg80Gln) and c.257_265delAGGCCATTG, p.(Glu86_Ile88del) in the same exon. The first variant has already been reported in literature as causative of 17 β-HSD3 deficiency.^11^ To the best of our knowledge, the second variant has not been identified before (Human Gene Mutation Database: https://www.hgmd.cf.ac.uk/ac/all.php; LOVD3 database: https://databases.lovd.nl/shared/variants/HSD17B3/unique) and was not found in gnomAD exomes database. In silico prediction tools indicated the pathogenicity of the variant. No pathogenetic variants were found in the other 19 genes of the panel.

## DISCUSSION

We here present the case of an additional Italian woman with 17 β-HSD3 deficiency, describing a novel pathogenetic variant in HSD17B3 gene. The extreme rarity of this condition is underlined by the fact that the multicentric evaluation conducted by the Italian DSD Study Group collected only 11 patients in Italy.^12^

The c.239G>A (rs119481075) is reported to be pathogenetic.^11^ In particular, this missense mutation results in an Arg to Gln substitution in exon 3 at position 80 p.(Arg80Gln), leading to a residual enzyme activity of 20%.^3^ The variant is reported in both ClinVar database (ID4874) and HGMD database (CM940950) and has been previously reported in homozygous and compound heterozygous state in multiple 46,XY individuals with pseudohermaphroditism and 17-beta hydroxysteroid dehydrogenase 3 deficiency, with virilization occurring at puberty.^8^^,^^13^ Furthermore, the other variant – which is not in a repeat region - results in a frame deletion of three aminoacids p.(Glu86_Ile88del). Given that the 17 β-HSD3 deficiency is an autosomal recessive DSD, the Authors assumed that the novel discovered variant in HSD17B3 gene is pathogenetic. Anyway, additional studies are needed to evaluate the way the c.257_265delAGGCCATTG is causative of enzyme deficiency and the related clinical phenotype. However, to date no correlation between phenotype and genotype has been described in 17 β-HSD3 deficiency.^4^ Since the patient's father has passed away years ago, it was not possible to perform DNA sequencing on the parents in order to establish whether the HSD17B3 variants are de novo mutations. Regarding clinical aspects of the present case, the patient was originally assigned female at birth due to the presence of female external genitalia and experienced virilization at puberty, as frequently reported in literature.^4^^,^^7^^,^^14^ The patient did not experience gender identity/gender role issues in late adolescence, even though a female to male gender role change is common in this DSD population, with an incidence ranging from 39%-64%.^4^ In order to rule out the possibility of testicular cancer and to avoid further pubertal changes, the patient underwent gonadectomy as well as clitoridectomy and vaginoplasty at the age of 16. That rather early intervention seemed reasonable at the time, given the female gender identity of the patient, even though she might have not been able to fully understand the implications of the surgical interventions. There is an intense debate about the proper time of sex assignment and reassignment in DSD individuals: according to the orientation of the most recent literature,^4^^,^^7^^,^^15^ irreversible interventions (such as genital surgery) should be performed only once full informed consent of the child is obtained. Some authors^4^ suggested a relatively early decision, since children with a female gender identity can experience virilization at puberty if gonadectomy is not performed promptly. However, other authors suggested the use of gonadotropin-releasing hormone analogues (GnRHa) in order to temporary suspend puberty in these adolescents gaining time to explore gender identity.^15^ A case-by-case evaluation is mandatory and the need to actively involve these individuals in the decision-making process appears crucial.^7^ In fact, in this case genital surgery performed at the same time of gonadectomy led the patient to a significative sexual discomfort in adult life, leading to a second intervention for clitoris reconstruction. This is in line with high rates of sexual dissatisfaction in individuals with 17beta HSD-3 deficiency in literature.^7^

The present case report has several limitations: first of all, the late referral of the patient to our center made it impossible to describe significant clinical aspects of the case (for example, no early hormonal work-up was available in the medical reports of the patient). Furthermore, to date we are not able to describe the pathogenetic mechanism of the novel c.257_265delAGGCCATTG variant described through the DNA sequencing of the patient.

On the other hand, we believe that the description of a novel mutation of the 17 β-HSD3 gene represents an important contribution to the process of investigation of a rare subtype of DSD such as 17beta HSD-3 deficiency.

## STATEMENT OF ETHICS

Written informed consent was obtained from the patient for publication of this case.

## STATEMENT OF AUTHORSHIP

C.C., A.D.F., J.R., L.V. and M.M; Acquisition of data: C.C., A.R., F.B., F.M. and A.D.F.; Analysis and interpretation of data: F.B.; Drafting the article: C.C., A.R., F.B., and F.M.; Revising it for intellectual content: J.R. and A.D.F; Final approval of the completed article: C.C., J.R, M.M., L.V. and A.D.F.
